# Preparation for shaddock skin polysaccharide derivatives by response surface method

**DOI:** 10.1038/s41598-024-63851-w

**Published:** 2024-06-18

**Authors:** Gangliang Huang, Bobo Lin

**Affiliations:** Key Laboratory of Carbohydrate Science and Engineering, Chongqing Normal University, Chongqing, 401331 China

**Keywords:** Preparation, Derivatives, Shaddock skin polysaccharide, Response surface method, Biochemistry, Biotechnology

## Abstract

The derivation of polysaccharide has an important impact on its properties. The preparation process of phosphorylated-shaddock skin polysaccharides (SSP) and acetylated-SSP was optimized by the response surface method. The constructed model was accurate and reliable in predicting the substitution of acetylated-SSP and the phosphate content of phosphorylated-SSP. This method was simple and easy to operate, which provided a basis for the preparation of a large number of derivatives.

## Introduction

Polysaccharide is a kind of biological macromolecule with multiple biological activities. Because of its advantages of safety and few side effects, polysaccharide is considered an ideal raw material in food and medicine. Polysaccharides are modified into derivatives with different structures, and there are significant differences in their biological activities. Among them, phosphorylation and acetylation are two important methods for polysaccharide modification^1–5^. Shaddock skin polysaccharides (SSP) have many physiological functions and have important application prospects for their research and development^6,7^. Herein, using SSP as the starting raw material, the preparation of their derivatives was studied. This would lay the foundation for the application of SSP.

## Experimental part

### Material

The shaddock skin polysaccharide was purchased from the market.

### Preparation of acetylated-SSP and optimization of process conditions

The effects of reaction time (1.0 h、1.5 h、2.0 h、2.5 h、3.0 h), the material ratio (m (SSP):v (acetic anhydride), 1:0.3, 1:0.6, 1:1.2, 1:1.8, 1:2.40) and reaction temperature (20 ℃、30 ℃、40 ℃、50 ℃、60 ℃) on the degree of acetylation substitution of acetylated-SSP were investigated by single-factor experiments. Based on the single-factor experiments, response surface optimization was carried out with SSP acetylation substitution degree as the response value, and the response time (A), material ratio (B) and reaction temperature (C) were used as the response variables for response surface optimization. The response surface test was designed with Design-Expert 8.0.6 software, and the quadratic regression orthogonal combination test with 3 factors and 3 levels was conducted (Table 1). The reaction was carried out.

**Table 1:** Box-Behnken test factors and levels of acetylated-SSP.

Level	Factor		
	A Reaction time (h)	B Material-liquid ratio	C Temperature (℃)
1	1.5	1:0.3	30
0	2.0	1:0.6	40
−1	2.5	1:1.2	50

### Preparation of phosphorylated-SSP and optimization of the preparation process

Single-factor experiments were conducted with the mass ratios of sodium tripolyphosphate and sodium trimetaphosphate (1:6, 2:5, 3:4, 4:3, 5:2, 6:1), reaction temperatures (30, 50, 70, 80, 90 °C) and reaction times (1, 2, 3, 4, 5, 6 h) as the investigating factors. Dimethyl sulfoxide (DMSO) was used as the solvent. Based on the single-factor experiments, response surface optimization was carried out with SSP phosphorylation substitution as the response value and phosphorylation reagent ratio (D), temperature (E) and time (F) as the response variables. The response surface test was designed by Design-Expert 8.0.6 software, and a quadratic regression orthogonal combination test with 3 factors and 3 levels was conducted (Table 2).

**Table 2:** Box-Behnken test factors and levels of phosphorylated-SSP.

Level	Factor		
	D phosphorylated reagent mass ratio	E temperature (℃)	F time (h)
1	4∶3	70	4
0	5∶2	80	5
−1	6∶1	90	6

## Results and discussion

### Single-factor experimental analysis

**Figure 1:**
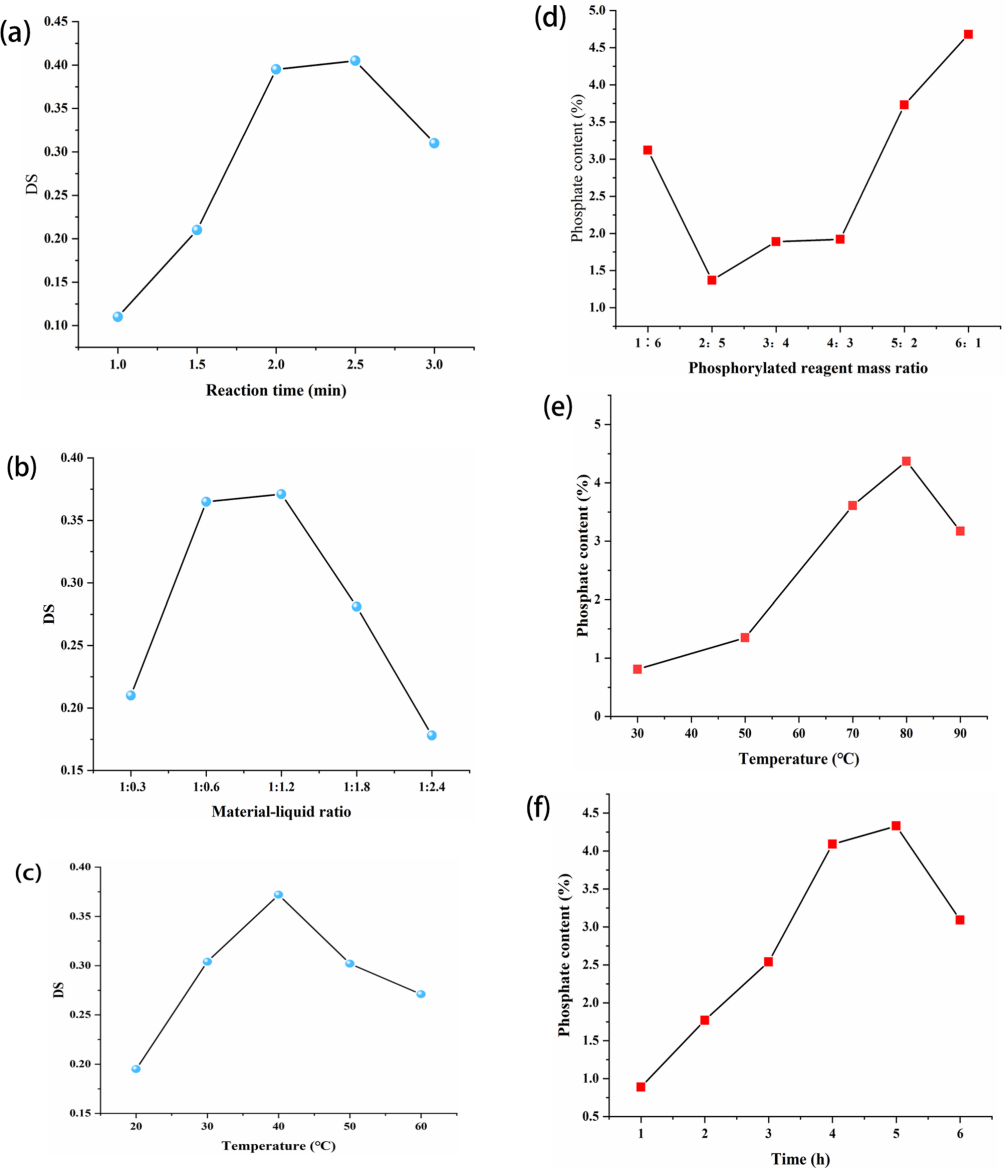
Effect of each single factor experiment on acetylated-SSP substitution and phosphorylated-SSP phosphate content.

From Fig. 1a, it could be learned that the degree of acetylation substitution showed an increasing trend when the reaction time was increased from 1.5 h to 3.0 h. However, when the reaction time reached 3 h, the degree of acetylation substitution showed a significant decreasing trend. It might be that with the increase of reaction time, the collision probability between the reactants increases, leading to the degradation of some polysaccharides, and thus affecting the degree of acetyl substitution of acetylated-SSP^8^. From Fig. 1b, it could be seen that the increase in the amount of acetic anhydride made the SSP dissolve more fully, and the constant contact between SSP and acetic anhydride eventually led to an increase in the degree of substitution, but when the percentage of acetic anhydride exceeded a certain range, the degree of acetyl substitution started to decrease, which was most likely due to the increase in the side reaction of acetic anhydride^9^. As shown in Fig. 1c, the degree of substitution tended to increase and then decrease as the reaction temperature increases. This trend might be because an appropriate temperature increase accelerated the raw material's swelling, allowing the polysaccharides and acetic anhydride to react more fully with each other. Therefore, when the temperature increased, the degree of acetyl substitution also increased. However, when the temperature was too high, the acetic anhydride in the reaction would be hydrolyzed and SSP could only react with part of the acetic anhydride, so the acetyl substitution decreased^10^.

From Fig. 1d, it can be learned that the phosphate content of phosphorylated-SSP did not change much when the ratio of sodium tripolyphosphate and sodium trimetaphosphate was 1:6 to 4:3. This is most likely because sodium tripolyphosphate and sodium trimetaphosphate have a mutually inhibiting effect. When the mass ratio of phosphorylation reagents was 4:3 to 6:1, the phosphate content of phosphorylated-SSP gradually increased with the increase of the proportion of sodium tripolyphosphate, which indicated that an appropriate increase of the proportion of sodium tripolyphosphate could promote phosphorylation. As shown in Fig. 1e, when the temperature is between 30℃ and 80℃, the content of the phosphate group increases gradually with the increase in temperature, which may be because the increase in temperature enhances the polymer bond activity of SSP^11^. From Fig. 1f, it is known that the phosphate content increased with increasing reaction time when the reaction time was from 1 to 5 h. This may be because the contact between the phosphorylation reagent and the SSP molecules was more adequate when the reaction time was properly extended, so it made the phosphate content increase. When the reaction time was between 5 and 6 h, the phosphate content showed a decreasing trend. It may be because the reaction time was too long, which leads to phenomena such as breakage or curling of the sugar chain of the polysaccharide^12,13^.

### Response surface analysis

#### Model fitting and statistical analysis

Based on the preliminary single-factor experimental results, the three parameters of reaction time, material ratio and reaction temperature were further investigated using response surface analysis to obtain the optimum acetylation conditions. The design matrix and corresponding experimental results are shown in Table 3, and the Design-Expert software (version 8.0.6) is used for data analysis. The response variable of the degree of acetylation substitution can be described by the following second-order polynomial equation:

**Table 3:** Three-variable encoding level and response value of SSP acetylation modification based on BBD.

Run	Reaction time (h)	Material-liquid ratio	Temperature (℃)	DS	
	A	B	C	Actual	Predicted
1	−1	−1	0	0.212	0.210
2	1	−1	0	0.343	0.339
3	−1	1	0	0.247	0.251
4	1	1	0	0.360	0.362
5	−1	0	−1	0.261	0.261
6	1	0	−1	0.379	0.381
7	−1	0	1	0.249	0.247
8	1	0	1	0.366	0.366
9	0	−1	−1	0.305	0.307
10	0	1	−1	0.374	0.374
11	0	−1	1	0.320	0.324
12	0	1	1	0.325	0.323
13	0	0	0	0.405	0.408
14	0	0	0	0.412	0.408
15	0	0	0	0.409	0.408
16	0	0	0	0.410	0.408
17	0	0	0	0.406	0.408

Degree of substitution =  + 0.41 + 0.060 * A + 0.016 * B − 7.375E-003 * C − 4.500E-003 * A * B − 2.500E-004 * A * C -0.016 * B * C − 0.068 * A^2^ − 0.050 * B^2^ − 0.027 * C^2^.

As can be seen from Table 4, F = 475.25 with a P-value < 0.0001, which indicates that the linear relationship between the dependent variable and the three independent variables is good and that the model reaches an extremely significant level. The misfit term of the model was used as a measure of the measurement data that it cannot represent within the experimental range, and the P-value for the misfit term in this experiment was 0.1325 > 0.05, which is insignificant compared to the pure error. This indicates that other factors have little effect on the acetylation substitution. The coefficient of variation of the equation CV = 1.18%, which indicates that the proportion of unexplained total variation is small, and the experimental value is reliable. The coefficient of determination R^2^ = 0.9984 and the correction coefficient Adj R-Squared = 0.9963 indicate a very good fit between the values predicted by the model and the actual experimental values (Table 5). All three independent variables examined in the model affected the degree of substitution of the SSP acetylation modification. The order of influence was A (reaction time) > B (material-liquid ratio) > C (reaction temperature). The effect of BC on the degree of substitution reached an extremely significant level in the interaction term, while the effect of AC and AB on the degree of substitution was not significant. In the quadratic term, the effect of the quadratic term on the degree of substitution for all three factors was at an extremely significant level.

**Table 4:** Response surface quadratic model ANOVA for SSP acetylation modification.

Source	Sum of squares	df	Mean square	F-value	P-value	Significant
Model	0.069	9	7.615 × 10^–3^	475.25	< 0.0001	⋆⋆
A-Reaction time	0.029	1	0.029	1789.98	< 0.0001	⋆⋆
B-Material-liquid ratio	1.985 × 10^–3^	1	1.985 × 10^–3^	123.86	< 0.0001	⋆⋆
C-Temperature	4.351 × 10^–4^	1	4.351 × 10^–4^	27.16	0.0012	⋆
AB	8.100 × 10^–5^	1	8.100 × 10^–5^	5.06	0.0593	
AC	2.500 × 10^–7^	1	2.500 × 10^–7^	0.016	0.9041	
BC	1.024 × 10^–3^	1	1.024 × 10^–3^	63.91	< 0.0001	⋆⋆
A^2^	0.019	1	0.019	1201.41	< 0.0001	⋆⋆
B^2^	0.011	1	0.011	666.59	< 0.0001	⋆⋆
C^2^	3.096 × 10^–3^	1	3.096 × 10^–3^	193.21	< 0.0001	⋆⋆
Residual	1.122 × 10^–4^	7	1.602 × 10^–5^			
Lack of Fit	8.075 × 10^–5^	3	2.692 × 10^–5^	3.43	0.1325	-
Pure Error	3.14 × 10^–5^	4	7.852 × 10^–6^			
Cor Total	0.069	16				

⋆⋆ means highly significant (P < 0.001), ⋆ means significant (P < 0.05).

– Means not significant.

**Table 5:** Reliability analysis of regression model.

Source		Source	
Std. Dev	4.003 × 10^–3^	R-Squared	0.9984
Mean	0.34	Adj R-Squared	0.9963
C.V. %	1.18	Pred R-Squared	0.9805
PRESS	1.341 × 10^–3^	Adeq Precision	64.529

Model fitting and statistical analysis were carried out using Design-Expert 8.0.6software to regress the phosphate content of the samples from the 17 specimen points obtained in Table 6 and the relationships between the response values and the factors were obtained as follows:

**Table 6:** Three-variable encoding level and response value of SSP phosphorylation modification based on BBD.

Run	Phosphorylated reagent mass ratio	Temperature (℃)	Time (h)	Phosphate content	
	D	E	F	Actual	Predicted
1	−1	0	1	2.350	2.380
2	0	0	0	4.231	4.170
3	0	0	0	4.230	4.170
4	0	0	0	4.070	4.170
5	1	0	−1	3.790	3.760
6	−1	0	1	4.210	4.170
7	1	1	0	3.170	3.164
8	0	−1	−1	3.870	3.896
9	0	0	0	4.110	4.170
10	−1	1	0	2.190	2.186
11	−1	−1	0	1.940	1.946
12	1	−1	0	3.770	3.7743
13	0	−1	1	3.470	3.434
14	0	1	−1	3.190	3.226
15	1	0	1	3.220	3.253
16	−1	0	−1	1.860	1.827
17	0	1	1	3.760	3.734

Phosphate content =  + 4.17 + 0.70 * A—0.092 * B + 0.011* C—0.21 * A * B − 0.27 * A * C + 0.24 * B * C − 1.09 * A^2^ -0.32 * B^2^ − 0.28 * C^2^.

The F-value and P-value can be used to test the significance of each coefficient and to assess the strength of the interaction between each factor. As can be seen from Tables 7 and 8, the F-value of the model is 274.83 with a P-value < 0.0001, which indicates that the regression model reaches an extremely significant level and that the linear relationship between the dependent variable and the three independent variables is good. In addition, the P-value for the misfit term was 0.7164 > 0.05, indicating that the difference between the model and the test values was small. The equation coefficient of variation CV = 1.96%, the coefficient of determination R^2^ = 0.9972 and the correction coefficient Adj R-Squared = 0.9935 indicate that the fit between the values predicted by the model and those of the actual test is excellent and adequate to represent the true relationship between the independent and response variables. The order of influence of the three independent variables examined in the model on the phosphate content of phosphorylated-SSP was D (phosphorylated reagent mass ratio) > E (temperature) > F (time). In the interaction term, the effect of DE on phosphate content reached an extremely significant level, while the effects of DF and EF on phosphate were not significant. Otherwise, the secondary terms of all three factors had an extremely significant level of effect on phosphate content.

**Table 7:** Response surface quadratic model ANOVA for SSP phosphorylation modifications.

Source	Sum of squares	df	Mean square	F-value	P-value	Significant
Model	10.79	9	1.20	274.83	< 0.0001	⋆⋆
D-Phosphorylated reagent mass ratio	3.93	1	33.93	901.53	< 0.0001	⋆⋆
F-Temperature	0.068	1	0.068	15.69	0.0055	
G-Time	1.01 × 10^–3^	1	1.012 × 10^–3^	0.23	0.6447	
DF	0.18	1	0.18	41.39	0.0004	
DE	0.28	1	0.28	64.37	< 0.0.0001	⋆⋆
EF	0.24	1	0.24	53.91	0.0002	
D^2^	4.96	1	4.96	1136.12	< 0.0001	⋆⋆
E^2^	0.42	1	0.42	97.33	< 0.0001	⋆⋆
F^2^	0.33	1	0.33	75.70	< 0.0001	⋆⋆
Residual	0.031	7	4.364 × 10^–3^			
Lack of Fit	8.025 × 10^–3^	3	2.675 × 10^–3^	0.48	0.7164	–
Pure Error	0.023	4	5.630 × 10^–3^			
Cor Total	10.82	16				

⋆⋆ means highly significant (P < 0.001), ⋆ means significant (P < 0.05).

– Means not significant.

**Table 8:** Reliability analysis of regression model.

Source		Source	
Std. Dev	0.066	R-Squared	0.9972
Mean	3.38	Adj R-Squared	0.9935
C.V. %	1.96	Pred R-Squared	0.9849
PRESS	0.16	Adeq Precision	46.240

#### Analysis of the interaction of the model factors

**Figure 2:**
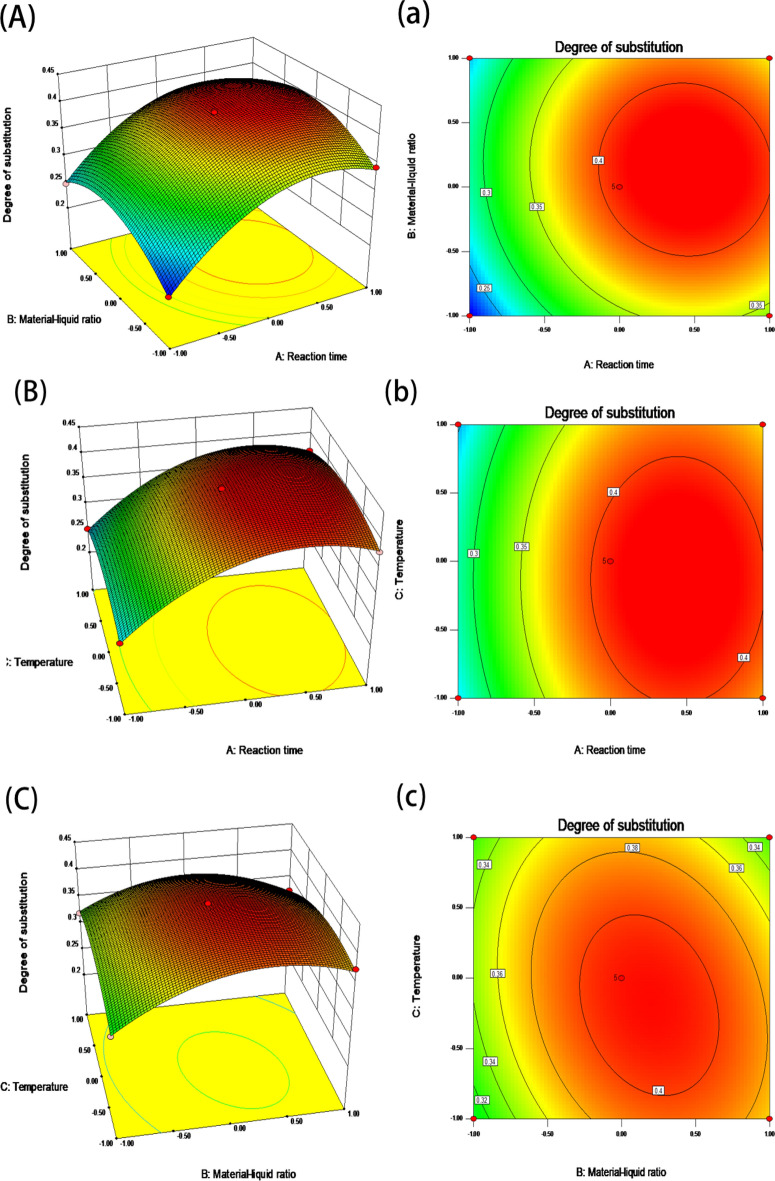

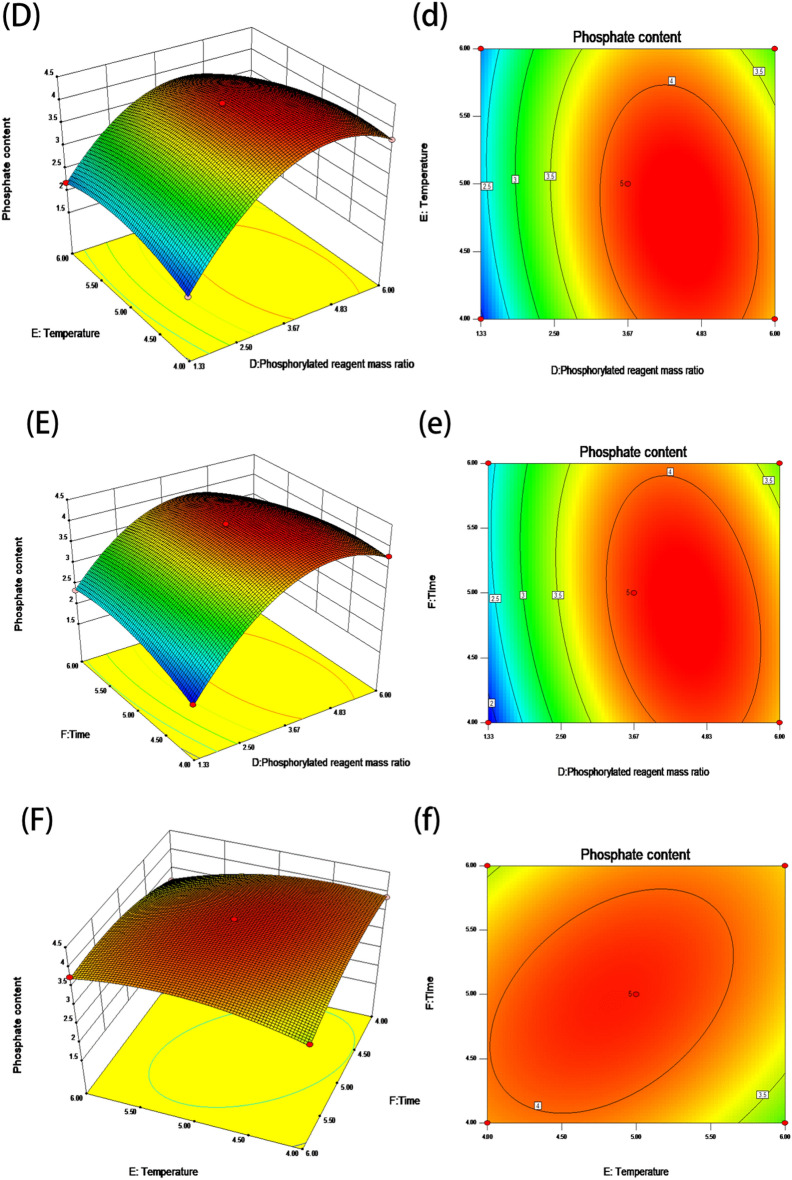
Response surface and contour plots of the interaction of the experimental factors.

The shape of the surface plots can be used to examine the important interaction of each element, and the slope degree of the surface has a positive correlation with significance^14^. If the response surface has a steeper slope and the contours are tight, it indicates that the interaction between the two factors has a significant effect on the response value^15^. If the surface is gentler and the contour lines are loose, it indicates that the interaction between the two factors has no significant effect on the response value. As can be seen from Fig. 2, the interaction between B and C, D and E forms the steepest slope of the surface, indicating that the interaction between the two groups of factors has the most significant effect on the response values. This is consistent with the ANOVA results.

### Determination and validation of optimum process conditions

The predicted optimal preparation solution based on the software and the actual measured results are shown in Tables 9 and 10.

**Table 9:** Validation of response surface results for acetylation modification.

	Reaction time (min)	Material-liquid ratio	Temperature (℃)	DS
Predicted value	2.30	1:0.96	36.18	0.412274
Actual value	2.0	1:1.2	40	0.426732

**Table 10:** Validation of response surface results for phosphorylation modification.

	Phosphorylated reagent mass ratio	Temperature (℃)	Time (h)	Phosphate content (%)
Predicted value	5.92:1	72.40	4.3	4.29252
Actual value	6:1	70	4	4.10531

After analysis, the difference between the predicted and actual results of acetylation substitution is only 1.4%, and the difference between the predicted and actual values of phosphate content is 0.187%, which proves that the constructed model is accurate and reliable in predicting the substitution of acetylated-SSP and the phosphate content of phosphorylated-SSP.

## Conclusion

The derivation of polysaccharides has an important impact on their properties. Using SSP as the starting raw material, the preparation method of SSP derivatives was systematically studied. At the same time, the response surface method was used to optimize the above preparation process and the expected effect was achieved. This preparation method provided a basis for the application of SSP.

## Data Availability

The datasets used and/or analysed during the current study available from the corresponding author on reasonable request.

## References

[1] Wang H, Huang G (2024). Extraction, purification, structural modification, activities and application of polysaccharides from different parts of mulberry. Food Funct..

[2] Yu B, Lin B, Huang G (2023). Preparation of acetylated grapefruit peel polysaccharide. Chem. Biodivers..

[3] Li J, Chen Z, Shi H, Yu J, Huang G, Huang H (2023). Ultrasound-assisted extraction and properties of polysaccharide from *Ginkgo biloba* leaves. Ultrason. Sonochem..

[4] Ji X, Cheng Y, Tian J, Zhang S, Shi M (2021). Structural characterization of polysaccharide from jujube (ziziphus jujuba mill.) fruit. Chem. Biol. Technol.Agric..

[5] Zhou S, Huang G (2021). Preparation, structure and activity of polysaccharide phosphate esters. Biomed. Pharmacother..

[6] Xu Y, Wu YJ, Sun PL, Zhang FM, Linhardt RJ, Zhang AQ (2019). Chemically modified polysaccharides: Synthesis, characterization, structure activity relationships of action. Int. J. Biol. Macromol..

[7] Xiao L, Ye F, Zhou Y, Zhao G (2021). Utilization of pomelo peels to manufacture value-added products: A review. Food Chem..

[8] Qi K, Xia G, Huang G, Huang H (2021). Extraction, chemical modification, and antioxidant activities of Daucus carota polysaccharide. Chem. Biol. Drug Des..

[9] Ren YY, Sun PP, Ji YP, Wang XT, Dai SH, Zhu ZY (2020). Carboxymethylation and acetylation of the polysaccharide from Cordyceps militaris and their α-glucosidase inhibitory activities. Nat. Prod. Res..

[10] Huang H, Huang G (2020). Extraction, separation, modification, structural characterization, and antioxidant activity of plant polysaccharides. Chem. Biol. Drug Des..

[11] Ji X, Guo J, Tian J, Ma K, Liu Y (2023). Research progress on degradation methods and product properties of plant polysaccharides. J. Light Ind..

[12] Xia S, Zhai Y, Wang X, Fan Q, Dong X, Chen M, Han T (2021). Phosphorylation of polysaccharides: A review on the synthesis and bioactivities. Int. J. Biol. Macromol..

[13] Yang W, Zhang Y, Tang A, Ruan Q, Huang G (2021). Preparation and antioxidant activity of phosphorylated polysaccharide from purple sweet potato. Chem. Biol. Drug. Des..

[14] Ryu DH, Cho JY, Sadiq NB, Kim JC, Lee B, Hamayun M, Lee TS, Kim HS, Park SH, Nho CW, Kim HY (2021). Optimization of antioxidant, anti-diabetic, and anti-inflammatory activities and ganoderic acid content of differentially dried Ganoderma lucidum using response surface methodology. Food Chem..

[15] Wang K, Li M, Wen X, Chen X, He Z, Ni Y (2018). Optimization of ultrasound-assisted extraction of okra (*Abelmoschus esculentus* (L.) Moench) polysaccharides based on response surface methodology and antioxidant activity. Int. J. Biol. Macromol..

